# Impact of the COVID-19 Pandemic on the Treatment of Head and Neck Cancers

**DOI:** 10.3390/jcm14051424

**Published:** 2025-02-20

**Authors:** Max L. Lee, Andrey Finegersh, Michelle M. Chen

**Affiliations:** 1 Department of Otolaryngology—Head and Neck Surgery, Stanford University School of Medicine, 801 Welch Road, Palo Alto, CA 94304, USA; maxlee12@stanford.edu (M.L.L.); afinegersh@stanford.edu (A.F.); 2 Department of Surgery, Palo Alto Veterans Administration, Palo Alto, CA 94304, USA; 3 ValleyCare Pleasanton Cancer Center, Pleasanton, CA 94588, USA

**Keywords:** squamous cell carcinoma of head and neck, COVID-19, pandemic, outcomes, disparities, otolaryngology

## Abstract

**Background/Objectives**: The COVID-19 pandemic led to unprecedented disruptions to cancer care, including the care of head and neck cancer. Given the necessity of timely treatment for mucosal cancers, it is important to understand how the pandemic affected the diagnosis, presentation, and treatment of mucosal head and neck cancer. **Methods**: The National Cancer Database was queried for patients with primary head and neck mucosal cancer. The number of annual diagnoses and the number of days between diagnosis and the start and end of any treatment were tracked over time from 2004 to 2020. Chi-square tests were used to compare differences in patient clinical and demographic characteristics in 2019 and 2020 to provide the most direct comparison. Multivariable linear regression and logic regression analyses were also used to compare three treatment quality measures between 2019 and 2020: number of days between diagnosis and start of treatment, number of days between surgery and start of postoperative radiation, and number of days between surgery and end of radiation. **Results**: The number of mucosal cancer diagnoses decreased (9.1%) during the early stages of the pandemic, with a larger decrease (12.4%) among patients receiving surgery. On multivariable analysis comparing 2020 to 2019, time to treatment was shorter (2.3 days; 95% CI, 1.69 to 2.85 days), and time from surgery to start of postoperative radiation was less likely to be delayed (OR, 0.91 of radiation greater than 42 days from surgery; 95% CI, 0.85 to 0.97). However, patients who were black, female, older, or uninsured were more likely to experience treatment delays. **Conclusions**: Overall, there were no treatment delays for patients with surgical head cancer and patients with neck cancer during the COVID-19 pandemic. However, vulnerable groups, such as patients who were black, female, older, and uninsured, were at higher risk of experiencing treatment delays.

## 1. Introduction

The impact of the early COVID-19 pandemic on in-person healthcare utilization has been well-documented [1,2,3], with decreases in emergency department utilization [1,4,5], inpatient hospital admissions [5,6], and cancer diagnoses [7,8]. Patients with cancer were particularly vulnerable to disruptions caused by the COVID-19 pandemic, given the importance of timely treatment [9] and increased risk of COVID-19-related complications and mortality [10,11]. Years later, research suggests patients were also at higher risk of developing longer-term complications, particularly long COVID-19, which include symptoms such as fatigue, cough, myalgias, and gastrointestinal symptoms [12].

In 2020, the pandemic led to significant delays in cancer screening, diagnosis, and treatment for many types of cancer [7,13]. Timely treatment is particularly important in patients with head and neck cancer (HNC) given there is a Commission on Cancer (CoC) quality metric associated with the time from surgery to initiation of radiation. Mucosal cancers in particular are associated with devastating complications, such as fatal bleeding, which require rigorous postoperative monitoring as well as multidisciplinary management, both of which were likely adversely affected by the COVID-19 pandemic [14]. Prior institutional studies have demonstrated that the number of patients evaluated and diagnosed with HNC in 2020 decreased, relative to 2019 [15,16,17,18]. There have been significant discrepancies in prior studies, where some did not find significant differences in patient characteristics, time to diagnosis, or time to treatment between patients diagnosed during the pandemic versus pre-pandemic [15,16], while other studies found significant delays [18]. Patients diagnosed with HNC in 2020 were also more likely to present with more advanced disease as measured by factors including tumor size, nodal disease, and staging [15,16,18,19,20].

Studies of the impact of the early COVID-19 pandemic on HNC treatment have primarily been conducted at individual academic centers over the span of a few months, resulting in small sample sizes with limited generalizability [15,16,17,18]. Moreover, none of these studies have evaluated the impact of the pandemic on the time from surgery to the initiation of radiation therapy. The goal of our study was to characterize differences in treatment times and clinical and demographic characteristics of patients diagnosed with HNC during the COVID-19 pandemic in the United States on a larger scale.

## 2. Methods

### 2.1. Data Source

The database used for this study was the National Cancer Database (NCDB), which captures over 70% of incident cases of cancer in the United States. This study was approved by the Institutional Review Board (IRB) of Stanford University School of Medicine, and the informed consent requirement was waived since the study was performed using a deidentified dataset.

### 2.2. Study Population and Covariates

#### 2.2.1. Classification of Mucosal Head and Neck Cancer

We identified patients from the NCDB from 2004 to 2020 who were diagnosed with primary HNC based on the International Classification of Diseases for Oncology, 3rd Edition (ICD-O3) diagnosis codes, which have remained unchanged throughout the time period of the study. Mucosal cancer subsites were divided into oral cavity, oropharynx (C019, C024, C051, C052, C090, C091, C098–C109, C140, and C142), hypopharynx (C129–C132, C138, and C139), and larynx (C320–C323, C328, and C329).

#### 2.2.2. Demographic and Clinical Variables

Patient demographic variables and clinical variables included age, sex, race, education, household income, distance from the treatment facility, insurance status, Charlson–Deyo score, tumor subsite, facility region, urban-rural classification, pathologic staging, and readmission. The percentage of adults without a high school education and median household income, by ZIP code of the patient’s residence, were stratified by quartiles of the US population and based on the US Census 2000. The 2010 and 2020 US Census data were not available in the NCDB dataset. Insurance status was classified into the following groups: Medicaid, Medicare, private insurance or managed care, other government, and uninsured or unknown. Rural–urban classification was grouped into metro, urban, rural, and not available or unknown. Facility regions were based on the US Census Bureau classifications [21].

#### 2.2.3. Cancer Staging, Treatment, and Clinical Outcomes

Clinical and pathologic TNM staging was classified according to the *8th Edition American Joint Committee on Cancer Staging Manual* (AJCC, Chicago, IL, USA) from 2018 to 2020 and according to the *Traditional AJCC Staging Manual* from 2004 to 2017 [22]. Readmission was categorized as an unplanned readmission within 30 days. Charlson–Deyo score was divided into two groups: scores of 0–1 and scores of 2–3.

Patients were also classified based on the types and combinations of treatment they received—surgery, radiation, and chemotherapy. Groups included surgery alone, surgery and adjuvant radiation, surgery and adjuvant chemoradiation, radiation alone, concurrent chemoradiation, or unknown. For example, a patient who received surgery but whose radiation and chemotherapy status was unknown was placed in the “unknown” group.

### 2.3. Statistical Analyses

#### 2.3.1. Descriptive Statistics

Inclusion criteria for descriptive statistics were all adult patients diagnosed with HNC between 2004 and 2020. The average annual percentage change (AAPC) and corresponding 95% confidence interval of the trend line were calculated between the years of 2004 and 2019 to track changes in mucosal cancer diagnoses, the number of days between surgery and start of any treatment, and the number of days between surgery and start of radiation over time. Additionally, 95% confidence intervals were calculated based on the annual percent change (APC) of these variables between 2004 and 2019 to give point estimates to determine whether the changes between 2019 and 2020 were anomalous. We opted for this method of showing changes over time because the NCDB only updates their statistics annually, making a more rigorous time-series analysis difficult to perform given the small number of data points. Chi-square tests were used to compare differences in patient demographic and clinical characteristics across years. To provide the most direct year-to-year analysis, we compared patients from 2019 to 2020.

#### 2.3.2. Regression Analyses

The regression analyses were limited to only patients diagnosed with HNC between 2019 and 2020 in order to compare 2020 directly to 2019. There were three quality metrics that we compared between 2019 and 2020 using multivariable regression analysis: number of days between diagnosis and start of treatment, number of days between surgery and start of radiation, and number of days between surgery and end of radiation. These quality measures have been shown to be associated with survival and outcomes in head and neck cancer [23,24,25].

To determine the relationship between the year and the number of days between diagnosis and start of treatment, we used multivariable linear regression, controlling for demographic and clinical characteristics. To determine the relationship between year and the number of days between surgery and the start of radiation, we used multivariable logistic regression, setting the cutoff for the binary variable at 42 days and controlling for demographic and clinical characteristics. This cutoff was determined using the National Comprehensive Cancer Network (NCCN) guidelines, which recommend that postoperative radiotherapy (PORT) for HNC is started within 6 weeks, or 42 days, of surgery [26,27]. To determine the relationship between year and the number of days between surgery and the end of radiation, we also used multivariable logistic regression, setting the cutoff for the binary variable at 100 days. This was determined based on evidence suggesting that the optimal treatment package time, time between surgery and completion of radiation, was less than 100 days [28]. All analysis was performed using STATA (Version 15.1, StataCorp LLC, College Station, TX, USA).

## 3. Results

### 3.1. Changes in HNC Diagnoses and Treatment over Time

**Figure 1:**
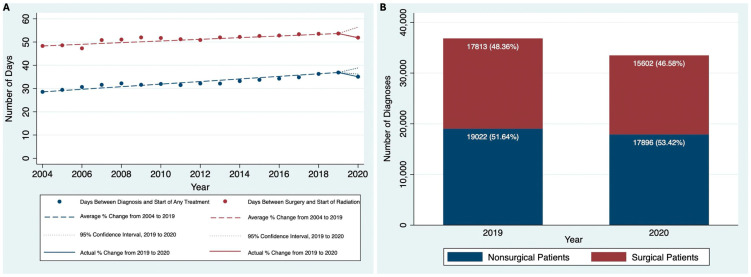
(**A**) Time to treatment quality metrics over time, 2004 to 2020, and (**B**) Surgical versus Nonsurgical patients (2019 and 2020).

Our cohort included 475,405 adult patients with HNC between 2004 and 2020. The AAPC in the number of HNC diagnosed between 2004 and 2019 was 4.2% (95% CI, 3.6% to 4.7%). However, between 2019 and 2020, the annual percent change (APC) in the number of HNC diagnoses was −9.1% (95% CI, 2.0% to 6.3%), representing a large drop in diagnoses from 37,005 to 33,652 that was significantly lower than what would be expected based on prior trends. For all of our quality metrics, we saw a similar pattern of a positive or flat trend from 2004 to 2019 followed by an abrupt negative trend between 2019 and 2020. Between 2004 and 2019, the AAPC in the number of days between diagnosis and start of treatment was 1.7% (95% CI, 0.8% to 2.6%). However, from 2019 to 2020, the APC was −4.9% (95% CI, −1.9% to 5.3%), which was lower than what would be expected based on prior trends (Figure 1A). For our time to radiation metrics, from 2004 to 2019, the AAPC in the number of days between surgery and start of radiation was flat at 0.72% (95% CI, −0.4% to 1.8%), and the APC from 2019 to 2020 was −3.3% (95% CI, −3.7% to 5.1%), which was not statistically significant (Figure 1A). Similarly, the AAPC for the number of days between surgery and completion of radiation from 2004 to 2019 was also flat at <0.1% (95% CI, −0.9% to 0.8%), and the APC between 2019 and 2020 was −2.2% (95% CI, −3.4% to 3.3%).

We also divided the cohort into surgical and nonsurgical patients and compared the AAPC in the number of diagnoses from 2004 to 2020. From 2004 to 2019, the AAPC in the number of diagnoses for the surgical group was 4.14% (95% CI, 2.96% to 5.32%), which was similar to the nonsurgical group at 4.19% (95% CI, 3.69% to 4.70%). However, from 2019 to 2020, the drop in the number of diagnoses was more in the surgical group (−12.41%, 95% CI of −0.1% to 8.8%) than the nonsurgical group (−5.92%, 95% CI of 2.2% to 6.2%) (Figure 1B) and was larger in both cases than what would be expected based on prior trends.

### 3.2. Demographic and Clinical Characteristics of Patients with HNC in 2019 and 2020

We compared patient characteristics between these two years for our primary analysis. The total cohort size for patients with HNC in 2019 and 2020 was 70,657 patients. Chi-square tests were used to compare patient demographic and clinical characteristics between patients diagnosed with HNC in 2019 versus 2020. Since the process of clinical and pathologic staging of cancers changed in 2018 with the new AJCC staging manual, patients in 2019 and 2020 were diagnosed using the same criteria. There were no clinically significant differences in patient demographic or clinical characteristics between patients diagnosed in 2019 versus 2020, although patients in 2020 were very slightly more likely to present with higher clinical T staging (33.32% cT3 and cT4 versus 30.74% cT3 and cT4) and pathologic T staging of disease (13.96% pT3 and pT4 versus 13.17% pT3 and pT4) versus patients in 2019 (Table 1). However, compared to patients diagnosed with HNC in 2019, patients diagnosed with HNC in 2020 were slightly more likely to be node positive at diagnosis (46.75% vs. 45.27%, *p* < 0.001) but did not have a difference in the rate of metastatic disease (3.51% vs. 3.29%, *p* = 0.250). It is important to note that a number of variables, particularly surgical margins and variables related to the pathologic staging of cancer, had a high proportion of patients classified as other or unknown. The reason there are more unknowns for pathologic staging and surgical margins is due to the fact that primary treatment for head and neck cancer can be definitive chemoradiation, definitive radiation, or surgery. For non-surgical patients, there would be no pathologic staging or surgical margins. In terms of clinical staging variables, there is a moderate proportion of unknown variables (11–21%), which is not unexpected. We know that there are patients with head and neck cancer of unknown primaries who have p16+/human-papillomavirus-associated disease, and often their primary is not identified, but are classified as patients with oropharyngeal cancer. This is a real-world study, and, as such, not all patients achieved complete nodal and distant metastatic staging, and those variables may be unknown.

### 3.3. Changes in Treatment Time Metrics from 2019 to 2020

After adjusting for clinical and socioeconomic characteristics, patients diagnosed in 2020 were found to have a shorter interval between diagnosis and start of treatment than those diagnosed in 2019 (mean difference = −2.27 days; 95% CI, −2.85 to −1.69 days). Other covariates that had a statistically significant association included race and distance from treatment facility. Black patients had to wait longer for treatment when compared to white patients (mean difference = 3.64 days; 95% CI, 2.51 to 4.77). Additionally, patients who lived further from the treatment facility had to wait longer for treatment, with patients who lived over 100 miles from the treatment facility waiting over a week longer (mean difference = 7.75 days; 95% CI, 6.31 to 9.18) than patients who lived 10 miles or fewer from the treatment facility (Table 2).

Patients with HNC in 2020 were less likely than patients in 2019 to have the time between surgery and the start of radiation be greater than 42 days (OR, 0.91; 95% CI, 0.85 to 0.97) and were less likely to have the time between surgery and the end of radiation be greater than 100 days (OR, 0.88; 95% CI, 0.83 to 0.93). Older patients were more likely to have a delay between surgery and the start of radiation. Female patients were more likely to have a delay between surgery and the start of radiation (OR: 1.18; 95% CI, 1.09 to 1.28) and were more likely to have a delay between surgery and the end of radiation (OR: 1.10; 95% CI, 1.03 to 1.18) relative to male patients. Compared with white patients, black patients were more likely to have a delay between surgery and the start of radiation (OR: 1.15; 95% CI, 1.01 to 1.32). Finally, patients who had either private insurance or managed care plans were less likely to have a delay between surgery and the start of radiation (OR: 0.73; 95% CI, 0.61 to 0.87) and a delay between surgery and the end of radiation (OR = 0.77; 95% CI, 0.66 to 0.89) relative to patients who were uninsured or whose insurance status was unknown (Table 3). Only clinical staging was used in this analysis since non-surgical patients did not have pathologic staging.

## 4. Discussion

We found a gradual increase in both HNC diagnoses and treatment times from 2004 to 2019, with abrupt decreases in both diagnoses and treatment times between 2019 and 2020 corresponding with the early stages of the COVID-19 pandemic. Patients in 2020 generally had similar demographic and clinical characteristics compared to patients in 2019 and were not more likely to present with distant metastases. After controlling for social and demographic characteristics, we found that patients treated during the pandemic were more likely to have shorter times from diagnosis to treatment as well as shorter times from the start of surgery to both the start and end of radiation treatment. As a whole, these differences were small, and our results suggest that hospitals were able to effectively prioritize multidisciplinary cancer treatment for HNC and avoid delays. Despite prioritization of cancer surgery during the pandemic, there were continued disparities, with race, gender, age, and insurance status being associated with delays in care.

From 2019 to 2020, there was a larger decrease in HNC surgical cases than in non-surgical cases. This is consistent with other studies that also found a more significant decrease in patients with surgical HNC [17,29,30]. An international study assessing 15 different cancer types, including HNC, cited lockdown measures and avoidance of COVID-19-related complications as potential reasons why surgeries were postponed, with health systems in lower-middle countries experiencing more significant delays [29]. However, another study conducted at an academic hospital found that while there were fewer cases performed, this was partially compensated for through a higher proportion of oncologic surgeries being performed, suggesting prioritization of serious cases [30]. Although the stage of disease at presentation was similar across the two years, there was a slightly higher proportion of cases that underwent non-surgical treatment (Table 1). Perhaps this was due to limitations in terms of capacity for surgery or treatment locally rather than at tertiary care centers.

Our study did not show any clinically meaningful difference in stage of presentation between 2019 and 2020. A large study performed in the Netherlands reported that there were no changes in tumor stage at presentation during the pandemic [31], while other smaller studies found an increase in patients presenting with higher stage HNC [15,16,19]. Of note, many of these studies are limited by small sample sizes and shorter time frames of only a few months, suggesting that there is likely wide geographical variation in delayed presentation of HNC [15,16,19]. As a whole, the data does not suggest that there was an obvious shift during the pandemic with decreased diagnosis of early-stage disease that may be found incidentally on imaging or during routine dental exams.

We found a consistent decrease in all three treatment time metrics during the pandemic. Prior United States studies have noted no increases or even slight decreases in time from diagnosis to treatment during the COVID-19 pandemic [15,32]. One reason for this is shorter waiting lists allowed patients to be treated in a more timely manner [31,33]. That being said, some international studies did demonstrate increased time from diagnosis to cancer treatment, citing COVID-19 lockdowns and risks concerning complications as potential contributing factors [29,34,35]. Facilities had a wide degree of variation in their ability to adapt cancer care to the pandemic. One study on breast cancer surgery in New York City public hospitals found that while the pandemic was not associated with delays to treatment overall, there was wide variation between treatment centers, with some hospitals successfully prioritizing cancer surgeries, leading to faster treatment times, while treatment at other hospitals ended up with significant delays [36].

Despite evidence suggesting that cancer centers were overall able to prioritize high-risk patients and prevent treatment delays, our study also found that older patients, female patients, black patients, and patients who were uninsured were more likely to have longer treatment times. Many of these patients are part of vulnerable populations who have historically faced barriers to accessing healthcare services even prior to the pandemic [37,38]. Studies suggest that many of these pre-existing health disparities were exacerbated for patients with cancer during COVID-19, leading to cancer treatment delays and higher rates of COVID-19-related complications and mortality among vulnerable groups [37,39,40]. Further work is necessary to develop targeted interventions and practices to support vulnerable patients during cancer care when similar situations arise in the future.

One important limitation of our study is the lack of information past 2020, making it difficult to assess the long-term impact of the pandemic on HNC treatment. Additionally, since the NCDB is only updated annually, we were limited from utilizing more rigorous time trend analysis to analyze the drop in diagnoses and increase in treatment times between 2019 and 2020. Therefore, although our study found that the drop in diagnoses between 2019 and 2020 was statistically significant, there is a possibility that the decrease in patient numbers could have been due to random variation. Another limitation of our dataset was that demographic characteristics, such as income and education quartiles, were referenced to the demographic characteristics of patients’ ZIP codes based on the 2000 US Census. Because demographic characteristics change significantly over time, this limits the accuracy of more recent data points. Additionally, the NCDB only contains facilities that are COC accredited, which biases the data towards cancer treatment centers that are likely better equipped to maintain high-quality cancer care during times of crisis, like the COVID-19 pandemic. This limits the generalizability of the study, considering the evidence suggesting that cancer treatment centers, particularly in lower-middle-income countries, had large variability in their ability to adapt cancer care to the pandemic, as well as our ability to calculate incidence rates [29,36]. Finally, the lack of data on disease-specific clinical outcomes such as complications and long-term survival makes it difficult to assess the impact of the COVID-19 pandemic on clinical care.

Our study demonstrated that there was no change in the clinical stage of patients who presented for care of HNC during the COVID-19 pandemic, and treatment time metrics were stable to decreased. Our findings suggest that hospitals were able to prioritize treatment of patients with HNC and avoid treatment delays. However, vulnerable groups, particularly patients who were black, female, elderly, or uninsured, were at higher risk of experiencing treatment delays. Further research is necessary to determine if there are any long-term ramifications to the COVID-19 pandemic in terms of the emergence of more advanced stage malignancies in the coming years.

## Figures and Tables

**Table 1:** Demographic and clinical characteristics, 2019 versus 2020.

	2019 N (%)	2020 N (%)	*p*-Value
**Age (in years)**			
≤50	3629 (9.81)	3311 (9.84)	*p* = 0.059
51–60	9799 (26.48)	8619 (25.61)	
61–70	12,853 (34.73)	11,776 (34.99)	
70+	10,724 (28.98)	9946 (29.56)	
**Sex**			
Male	27,413 (74.08)	24,814 (73.74)	*p* = 0.233
Female	9590 (2592)	8838 (26.26)	
**Race**			
White	31,748 (85.79)	28,856 (85.75)	*p* = 0.068
Black	3443 (9.30)	3035 (9.02)	
Other	1814 (4.90)	1761 (5.23)	
**Proportion of adults from patient’s ZIP code not graduating high school, 2000 US Census data**			
29.0%	5409 (17.66)	4773 (17.22)	*p* = 0.097
20% to 28.9%	7514 (24.54)	6876 (24.80)	
14% to 19.9%	7315 (23.89)	6818 (24.60)	
Less than 14%	10,387 (33.92)	9254 (33.38)	
**Median household income for patient’s ZIP code, 2000 US Census data**			
<USD 30,000	4421 (14.43)	3873 (13.97)	*p* = 0.075
USD 30,000–USD 34,999	5695 (18.59)	5327 (19.21)	
USD 35,000–USD 45,999	8692 (28.38)	7969 (28.74)	
USD 46,000+	11,823 (38.60)	10,555 (38.07)	
**Distance from facility (miles)**			
≤10	12,762 (34.66)	11,607 (34.65)	*p* = 0.639
11 to 20	6420 (17.44)	5937 (17.72)	
21 to 50	7008 (19.03)	6293 (18.78)	
51 to 100	3087 (8.38)	2872 (8.57)	
>100	7545 (20.49)	6792 (20.27)	
**Insurance status**			
Uninsured	1559 (4.32)	1367 (4.06)	*p* = 0.302
Private Insurance/ Managed Care	12,898 (34.85)	11,656 (34.64)	
Medicaid	3951 (10.68)	3675 (10.92)	
Medicare	17,456 (47.17)	15,982 (47.49)	
Other	1101 (2.98)	972 (2.89)	
**Charlson–Deyo score**			
0–1	33,034 (89.27)	29,904 (88.86)	*p* = 0.084
2–3	3971 (10.73)	3748 (11.14)	
**Primary site**			
Oral Cavity	11,250 (30.40)	10,246 (30.45)	*p* = 0.916
Oropharynx	15,625 (42.22)	14,260 (42.37)	
Hypopharynx	1607 (4.34)	1432 (4.26)	
Larynx	8523 (23.03)	7714 (22.92)	
**Readmission**			
No unplanned readmission	35,990 (97.26)	32,811 (97.50)	*p* < 0.001 *
Unplanned readmission	628 (1.70)	583 (1.73)	
Unknown	387 (1.05)	258 (.77)	
**Surgical margins**			
Negative	13,569 (36.67)	11,954 (35.52)	***p* = 0.001**
Positive	2550 (6.89)	2241 (6.66)	
Unknown	20,886 (56.44)	19,457 (57.82)	
**Facility region**			
East	7189 (19.43)	6208 (18.45)	***p* = 0.001**
South	9093 (24.57)	8518 (25.31)	
Midwest	14,582 (39.41)	13,429 (39.91)	
West	5371 (14.51)	4869 (14.47)	
Unknown	770 (2.08)	628 (1.87)	
**Rural–urban**			
Metro	28,899 (78.09)	26,417 (78.50)	***p* < 0.001**
Urban	6116 (16.53)	5666 (16.84)	
Rural	792 (2.14)	678 (2.01)	
Unknown	1198 (3.24)	891 (2.65)	
**Treatment**			
Surgery Only	9614 (25.98)	8306 (24.68)	***p* < 0.001**
Surgery and Radiation	4147 (11.21)	3811 (11.32)	
Surgery and Chemoradiation	3490 (9.43)	2924 (8.69)	
Radiation Only	4496 (12.15)	4415 (12.23)	
Chemoradiation Only	10,594 (28.63)	9862 (29.31)	
One or More Factors Unknown	4664 (12.60)	4634 (13.77)	
**Pathologic T stage**			
T1	5521 (14.92)	4475 (13.30)	***p* < 0.001**
T2	3738 (10.10)	3399 (10.10	
T3	1994 (5.39)	1867 (5.55)	
T4	2878 (7.78)	2828 (8.41)	
Other/Unknown	22,873 (61.81)	21,071 (62.64)	
**Pathologic N stage**			
N0	5340 (14.43)	4881 (14.50)	*p* = 0.839
N+	5923 (16.01)	5334 (15.85)	
Other/Unknown	25,742 (69.56)	23,437 (69.65)	
**Pathologic M stage**			
M0	16,185 (43.74)	14,203 (42.21)	***p* < 0.001**
M+	434 (1.17)	406 (1.21)	
Other/Unknown	20,386 (55.09)	19,043 (56.59)	
**Clinical T stage**			
T1	7968 (23.25)	6800 (21.97)	***p* < 0.001**
T2	8576 (25.02)	7642 (24.69)	
T3	5381 (15.70)	5262 (17.00)	
T4	5157 (15.04)	5051 (16.32)	
Other/Unknown	7196 (20.99)	6193 (20.01)	
**Clinical N stage**			
N0	15,108 (40.83)	13,255 (39.39)	***p* < 0.001**
N+	16,752 (45.27)	15,733 (46.75)	
Other/Unknown	5145 (13.90)	4664 (13.86)	
**Clinical M stage**			
M0	31,555 (85.27)	28,662 (85.17)	*p* = 0.250
M+	1216 (3.29)	1180 (3.51)	
Other/Unknown	4234 (11.44)	3810 (11.32)	

* *p*-values < 0.05 are shown in bold font.

**Table 2:** Multivariable linear regression analysis of time from surgery to the initiation of treatment, 2019 vs. 2020.

Independent Variable	Mean Difference (Days)	CI	*p*-Value
**Year 2020 (ref: Year 2019)**	−2.27	−2.85 to −1.69	<0.001 *
**Age, y (ref: ≤50 y)**			
51–60 y	1.99	0.80 to 3.18	**0.001**
61–70 y	1.98	0.78 to 3.18	**0.001**
71 y or older	0.31	−1.01 to 1.64	0.646
**Sex (ref: Male)**	1.39	0.68 to 2.11	**<0.001**
**Race (ref: White)**			
Black	3.64	2.51 to 4.77	**<0.001**
Other	0.84	−0.63 to 2.31	0.265
**Proportion of adults from patient’s ZIP code not graduating high school, 2000 US Census data (ref: 29.0%+)**			
20% to 28.9%	−0.35	−1.43 to 0.72	0.518
14% to 19.9%	−0.27	−1.45 to 0.92	0.658
Less than 14%	−1.49	−2.78 to −0.21	**0.023**
**Median household income for patient’s ZIP code, 2000 US Census data (ref: <USD 30,000)**			
USD 30,000–USD 34,999	−1.08	−2.23 to 0.61	0.064
USD 35,000–USD 45,999	−0.86	−2.05 to 0.33	0.158
USD 46,000+	−2.03	−3.35 to −0.70	**0.003**
**Distance from treatment facility (ref: 0–10 miles away from treatment facility)**			
11–20 miles	1.12	−0.33 to 1.91	**0.005**
21–50 miles	2.13	1.30 to 2.96	**<0.001**
51–100 miles	3.66	2.49 to 4.83	**<0.001**
>100 miles	7.75	6.31 to 9.18	**<0.001**
**Insurance status (ref: uninsured or unknown)**			
Private Insurance or Managed Care	−5.81	−7.57 to −4.05	**<0.001**
Medicaid	1.99	−0.02 to 4.00	0.053
Medicare	−3.47	−5.24 to −1.69	**<0.001**
Other Government	0.20	−2.25 to 2.66	0.871
**Charlson–Deyo score of 2 or 3 (ref: score of 0 or 1)**	0.31	−0.65 to 1.27	0.533
**Tumor site (ref: oral)**			
Oropharynx	−10.31	−11.24 to −9.37	**<0.001**
Hypopharynx	−10.05	−11.57 to −8.53	**<0.001**
Larynx	−9.46	−10.43 to −8.49	**<0.001**
**Facility region (ref: east)**			
South	−2.85	−3.71 to −1.99	**<0.001**
Midwest	0.02	−0.83 to 0.87	0.957
West	1.39	0.31 to 2.48	**0.012**
Unknown	−4.54	−6.73 to −2.34	**<0.001**
**Rural/urban (ref: metro)**			
Urban	−0.13	−1.08 to 0.83	0.793
Rural	−4.09	−5.96 to −2.22	**<0.001**
Not available/Unknown	−4.52	−6.21 to −2.83	**<0.001**
**Clinical T stage (ref: T1)**			
T2	4.88	4.05 to 5.72	**<0.001**
T3	7.84	6.84 to 8.84	**<0.001**
T4	9.43	8.35 to 10.52	**<0.001**
Other/Unknown	−0.82	−1.84 to 0.21	0.118
**Clinical N stage (ref: N0)**			
N+	−2.99	−3.82 to −2.17	**<0.001**
Other/Unknown	−2.81	−4.72 to −0.91	**0.004**
**Clinical M stage (ref: M0)**			
M+	−2.30	−4.21 to −0.39	**0.018**
Other/Unknown	−5.29	4.18 to 8.68	**<0.001**
**Treatment (ref: surgery only)**			
Surgery and Adjuvant Radiation	−3.09	−4.09 to −2.08	**<0.001**
Surgery and Adjuvant Chemoradiation	−2.75	−3.90 to −1.60	**<0.001**
Radiation only	16.86	15.70 to 18.02	**<0.001**
Definitive Chemoradiation	10.29	9.22 to 11.37	**<0.001**
Unknown	11.18	9.34 to 13.01	**<0.001**

CI: 95% confidence interval; * Significant *p*-values (<0.05) are shown in bold font.

**Table 3:** Multivariable logistic regression of time from surgery to the start and end of radiation, 2019 vs. 2020.

	Time from Surgery to Start of Radiation			Time from Surgery to End of Radiation		
Independent Variable	Odds Ratio	95% Confidence Interval	*p*-Value	Odds Ratio	95% Confidence Interval	*p*-Value
**Year 2020 (ref: Year 2019)**	0.91	0.85 to 0.97	0.004 *	0.88	0.83 to 0.93	<0.001 *
**Age, y (ref: ≤50 y)**						
51–60 y	1.16	1.03 to 1.30	**0.011**	1.23	1.11 to 1.36	**<0.001**
61–70 y	1.26	1.12 to 1.42	**0.014**	1.35	1.22 to 1.50	**<0.001**
71 y or older	1.63	1.42 to 1.87	**<0.001**	1.71	1.52 to 1.92	**<0.001**
**Sex (ref: Male)**	1.18	1.09 to 1.28	**<0.001**	1.10	1.03 to 1.18	**0.003**
**Race (ref: White)**						
Black	1.15	1.01 to 1.32	**0.039**	1.08	0.96 to 1.21	0.205
Other	0.97	0.84 to 1.12	0.689	0.96	0.85 to 1.08	0.474
**Proportion of adults from patient’s ZIP code** **not graduating high school, 2000 US Census** **Data (ref: 29.0%+)**						
20% to 28.9%	1.02	0.91 to 1.15	0.720	1.01	0.91 to 1.11	0.904
14% to 19.9%	0.93	0.82 to 1.06	0.271	0.98	0.88 to 1.09	0.724
Less than 14%	0.90	0.79 to 1.03	0.122	0.94	0.84 to 1.05	0.269
**Median household income for patient’s ZIP code,** **2000 US Census data (ref: <USD 30,000)**						
USD 30,000–USD 34,999	0.86	0.76 to 0.98	**0.028**	0.85	0.76 to 0.95	**0.005**
USD 35,000–USD 45,999	0.87	0.76 to 0.99	**0.038**	0.81	0.72 to 0.91	**<0.001**
USD 46,000+	0.84	0.72 to 0.97	**0.019**	0.73	0.64 to 0.83	**<0.001**
**Distance from treatment facility (ref: 0–10 miles)**						
11–20 miles	0.98	0.90 to 1.07	0.684	1.02	0.94 to 1.10	0.627
21–50 miles	1.25	1.14 to 1.38	**<0.001**	1.23	1.14 to 1.33	**<0.001**
51–100 miles	1.35	1.19 to 1.54	**<0.001**	1.41	1.26 to 1.57	**<0.001**
>100 miles	1.11	0.96 to 1.28	0.162	1.28	1.13 to 1.45	**<0.001**
**Insurance status (ref: uninsured/unknown)**						
Private Insurance or Managed Care	0.73	0.61 to 0.87	**<0.001**	0.77	0.66 to 0.89	**0.001**
Medicaid	1.25	1.02 to 1.54	**0.031**	1.27	1.07 to 1.51	**0.006**
Medicare	0.88	0.73 to 1.05	0.161	0.93	0.80 to 1.09	0.365
Other Government	1.02	0.78 to 1.34	0.869	1.07	0.85 to 1.34	0.581
**Charlson–Deyo score of 2–3 (ref: score of 0–1)**	1.17	1.04 to 1.31	**0.011**	1.18	1.07 to 1.31	**0.001**
**Tumor site (ref: Oral Cavity)**						
Oropharynx	0.47	0.43 to 0.51	**<0.001**	0.58	0.53 to 0.62	**<0.001**
Hypopharynx	0.69	0.54 to 0.88	**0.002**	0.86	0.70 to 1.05	0.150
Larynx	0.42	0.38 to 0.47	**<0.001**	0.55	0.50 to 0.60	**<0.001**
**Readmission (ref: no unplanned readmission)**						
Unplanned Readmission	1.33	1.05 to 1.68	**0.016**	1.31	1.08 to 1.59	**0.005**
Unknown	0.73	0.53 to 0.99	**0.042**	0.73	0.55 to 0.97	**0.033**
**Facility region (ref: east)**						
South	0.75	0.68 to 0.83	**<0.001**	0.89	0.82 to 0.97	**0.006**
Midwest	0.89	0.80 to 0.98	**0.017**	1.13	1.04 to 1.23	**0.004**
West	0.91	0.81 to 1.02	0.108	0.99	0.90 to 1.09	0.772
Unknown	0.84	0.67 to 1.06	0.144	0.99	0.82 to 1.20	0.908
**Rural/urban (ref: metro)**						
Urban	0.90	0.81 to 1.01	0.062	0.86	0.78 to 0.94	**0.001**
Rural	0.86	0.67 to 1.11	0.255	0.83	0.67 to 1.03	0.087
Not available/Unknown	1.07	0.88 to 1.30	0.477	0.99	0.84 to 1.17	0.931
**Pathologic T stage (ref: stage 1)**						
T2	0.78	0.71 to 0.86	**<0.001**	0.63	0.58 to 0.69	**<0.001**
T3	0.61	0.54 to 0.69	**<0.001**	0.43	0.39 to 0.48	**<0.001**
T4	0.65	0.58 to 0.74	**<0.001**	0.43	0.39 to 0.48	**<0.001**
Other/Unknown	3.89	3.30 to 4.58	**<0.001**	3.83	3.33 to 4.40	**<0.001**
**Pathologic N stage (ref: N0)**						
N+	0.41	0.37 to 0.45	**<0.001**	0.37	0.34 to 0.40	**<0.001**
Other/Unknown	0.75	0.67 to 0.84	**<0.001**	1.05	0.96 to 1.15	0.305
**Pathologic M stage (ref: M0)**						
M+	3.39	2.35 to 4.91	**<0.001**	4.29	3.14 to 5.87	**<0.001**
Other/Unknown	1.57	1.34 to 1.85	**<0.001**	1.31	1.15 to 1.50	**<0.001**

CI: 95% confidence interval; * Significant *p*-values (<0.05) are shown in bold font.

## Data Availability

The data presented in this study are provided by the American College of Surgeons, by application.

## Abbreviations

The following abbreviations are used in this manuscript:

HNC	Head and Neck Cancer
NCDB	National Cancer Database
IRB	Institutional Review Board
AJCC	American Joint Committee on Cancer
APC	Average Percentage Change
CI	Confidence Intervals
NCCN	National Comprehensive Cancer Network
PORT	Postoperative Radiotherapy
AAPC	Average Annual Percentage Change
